# Outcome and management of decompressive hemicraniectomy in malignant hemispheric stroke following cardiothoracic surgery

**DOI:** 10.1038/s41598-023-40202-9

**Published:** 2023-08-10

**Authors:** Peter Truckenmueller, Jonas Fritzsching, Daniel Schulze, Anton Früh, Stephan Jacobs, Robert Ahlborn, Peter Vajkoczy, Vincent Prinz, Nils Hecht

**Affiliations:** 1 Department of Neurosurgery and Center for Stroke Research Berlin (CSB), Charité – Universitätsmedizin Berlin, Campus Benjamin Franklin, Hindenburgdamm 30, 12203 Berlin, Germany; 2 Department of Cardiothoracic and Vascular Surgery, Deutsches Herzzentrum Berlin, Berlin, Germany; 3 Institute of Medical Biometrics and Clinical Epidemiology, Charité – Universitätsmedizin Berlin, Berlin, Germany; 4 Institute of Medical Informatics, Charité – Universitätsmedizin Berlin, Berlin, Germany; 5 Department of Neurosurgery, Goethe Universität Frankfurt, Frankfurt am Main, Germany

**Keywords:** Stroke, Cardiac device therapy, Comorbidities

## Abstract

Management of malignant hemispheric stroke (MHS) after cardiothoracic surgery (CTS) remains difficult as decision-making needs to consider severe cardiovascular comorbidities and complex coagulation management. The results of previous randomized controlled trials on decompressive surgery for MHS cannot be generally translated to this patient population and the expected outcome might be substantially worse. Here, we analyzed mortality and functional outcome in patients undergoing decompressive hemicraniectomy (DC) for MHS following CTS and assessed the impact of perioperative coagulation management on postoperative hemorrhagic and cardiovascular complications. All patients that underwent DC for MHS resulting as a complication of CTS between June 2012 and November 2021 were included in this observational cohort study. Outcome was determined according to the modified Rankin Scale (mRS) score at 1 and 3–6 months. Clinical and demographic data, anticoagulation management and postoperative hemorrhagic and thromboembolic complications were assessed. In order to evaluate a predictive association between clinical and radiological parameters and the outcome, we used a multivariate logistic regression analysis. Twenty-nine patients undergoing DC for MHS after CTS with a female-to-male ratio of 1:1.9 and a median age of 60 (IQR 49–64) years were identified out of 123 patients undergoing DC for MHS. Twenty-four patients (83%) received pre- or intraoperative substitution. At 30 days, the in-hospital mortality rate and neurological outcome corresponded to 31% and a median mRS of 5 (5–6), which remained stable at 3–6 months [Mortality: 42%, median mRS: 5 (4–6)]. Postoperatively, 15/29 patients (52%) experienced new hemorrhagic lesions and Bayesian logistic regression predicting mortality (mRS = 6) after imputing missing data demonstrated a significantly increased risk for mortality with longer aPPT (OR = 13.94, p = .038) and new or progressive hemorrhagic lesions after DC (OR = 3.03, p = .19). Notably, all but one hemorrhagic lesion occurred before discontinued anticoagulation and/or platelet inhibition was re-initiated. Despite perioperative discontinuation of anticoagulation and/or platelet inhibition, no coagulation-associated cardiovascular complications were noted. In conclusion, Cardiothoracic surgery patients suffering MHS will likely experience severe neurological disability after DC, which should remain a central aspect during counselling and decision-making. The complex coagulation situation after CTS, however, should not per se rule out the option of performing life-saving surgical decompression.

## Introduction

Ischemic stroke remains a frequent and devastating complication after cardiothoracic surgeries (CTS) leading to excess mortality and morbidity^1,2^. Analyses from databases and observational registers suggest an overall incidence between 0.98 and 5.2%^2,3^. In patients with type A acute aortic dissection (TAAAD), the rate of perioperative stroke can reach up to 32%^4,5^ and the in-hospital mortality rate in patients suffering from a perioperative stroke was significantly higher than in patients without stroke (32.8% vs. 4.9%^1^ or 14.93% vs. 2.15%^6^). The pathophysiological mechanisms of perioperative strokes primarily include thromboembolism and extension of an aortic dissection into the carotid artery and might cause a large vessel occlusion with subsequent malignant hemispheric stroke (MHS)^7^. This subtype of ischemic stroke may account for the majority of the morbidity and mortality associated with perioperative strokes. However, despite multiple studies on the incidence of perioperative cerebral stroke, there is only limited data on the number and outcome of patients suffering MHS following CTS^3,8^.

Neurosurgical management of patients with MHS after CTS poses a major dilemma, since CTS in the context of a life-threatening emergency predisposes patients to an increased risk for postoperative complications and morbidity^9^. This is highly relevant in the context of interdisciplinary decision making, since patients with such severe cardiovascular comorbidities were not included in previously published trials on MHS and the results of those randomized controlled trials cannot be generally translated to this patient population^10–14^. The expected functional outcome might be substantially worse, but so far, there is no systematic information addressing this issue and the lack of appropriate evidence hampers clinical decision-making and patient counselling. Further, decision-making in the setting of MHS after CTS is complicated by conflicting requirements for perioperative coagulation management, because antiplatelet and anticoagulant therapy is a central component of the patient management after CTS^15^. Therefore, we analyzed the clinical outcome, coagulation management and its impact on hemorrhagic and thromboembolic complications in patients undergoing DC for treatment of MHS following CTS in two of Europe’s highest-volume neurosurgical and cardiothoracic departments.

## Methods

### Study design

This observational cohort study was approved by the ethics committee of the Charité – Universitätsmedizin Berlin (EA4/019/22) and performed in compliance with Health Insurance Portability and Accountability Act regulations. For this study informed consent has been waived by the Charité – Universitätsmedizin Berlin ethics committee due to the anonymity and retrospective nature of the study. Data acquisition and presentation was done according to the STROBE guidelines for reporting observational studies^16^. All authors had full access to all the data in the study and take responsibility for its integrity and analysis.

### Patient management

We identified 282 patients who underwent DC at the Department of Neurosurgery at the Charité Virchow Campus in Berlin, Germany, between June 2012 and November 2021. Of these, 123 underwent DC for MHS and 29/123 (24%) underwent DC for MHS following CTS in the German Heart Center Berlin, Germany (DHZB; Deutsches Herzzentrum Berlin). Malignant hemispheric stroke with a volume ≥ 140 cm^3^ was detected by computerized tomography (CT) due to delayed recovery or new neurological impairment after withdrawal of anesthesia following CTS. The decision to perform DC was primarily based on the uniform inclusion criteria of the four randomized controlled trials on hemicraniectomy, DECIMAL, DESTINY I/II and HAMLET^10–14^. Exclusion criteria, such as pre-morbid mRS > 1, pre-morbid Barthel-Index < 95, other concomitant severe disease that would confound with treatment, or life expectancy < 3 years were not applied. DC was performed as previously described^17,18^. All patients were anaesthetized with propofol and remifentanil and received a bodyweight-adapted dose of 50 mg/kg mannitol 30 min before skin incision. Mean arterial blood pressure was targeted at 80–90 mmHg. The end-expiratory carbon dioxide concentration during surgery was maintained at a level corresponding to an arterial partial pressure of carbon dioxide between 38 and 42 mmHg. Postoperatively, patients were transferred to our neurointensive care unit. Intracranial pressure (ICP) was continuously monitored and patients remained intubated and sedated until ICP was within normal ranges. A critical ICP threshold was defined as ICP > 20 mmHg for longer than 10 min and treated with cerebrospinal fluid drainage, osmotic therapy, deep sedation and cerebrospinal fluid drainage, if an EVD was inserted: According to our protocol, we always aim to insert an EVD instead of an ICP probe as a first line measure in patients that require ICP monitoring. In patients suffering MHS, however, placement of an EVD/ICP probe prior to the surgery is usually not performed, because the indication for DC in MHS is usually not based on an ICP threshold. Further, the majority of the patients in our cohort that required postoperative ICP monitoring primarily received an ICP probe instead of an EVD, because intraoperative placement of a parenchymal ICP probe is likely easier than attempting to correctly place an EVD due to the compression and lateralization of the ventricles.

A routine postoperative CT was performed within 24 h after surgery. Infarct volumetry was done on postoperative CT scans performed within 24 h after DC using Brainlab Cranial Planning SmartBrush Ver. 2.6.0.121 (Brainlab AG, Munich, Germany). Volume gain was calculated as ipsilateral volume minus contralateral volume. Swelling was determined as volume gain divided by contralateral volume. Volume of infarction corrected for hemispheric swelling was calculated as volume of infarction divided by swelling plus 1.

### Outcome parameters

Demographic, clinical and radiographic patient data were retrospectively analyzed by a clinician who was not directly involved in the patients’ care. Primary endpoint was the functional status expressed on the modified Rankin Scale (mRS) score assessed at 1 and 3–6 months after DC. Since only one patient achieved a favorable functional outcome with an mRS score of ≤ 3, outcome was dichotomized into mRS 6 (death) versus mRS < 6 in the predictive models to assess variables associated with a higher risk for mortality. The secondary endpoint was postoperative occurrence of new hemorrhagic lesions. For this purpose, we analyzed all available postoperative CT scans regarding hemorrhagic lesions including hemorrhagic transformation within the established ischemic lesion, subgaleal hemorrhage, and intracerebral hemorrhage (ICH) beyond the established ischemic lesion.

### Statistical analysis

Statistics were calculated using GraphPad Prism version 9 (GraphPad Software Inc, San Diego, CA, USA) and R version 4.2.2 (R Foundation for Statistical Computing, Vienna, Austria, 2022). Variables are reported as median with interquartile range (IQR). In order to evaluate a predictive association between clinical and radiological parameters and outcome, we used a multivariate logistic regression analysis that included the following variables: age, sex, infarct volume, type of surgery [(TA(B)AAD or valve surgery], whether CTS was an emergency or elective surgery, left affected hemisphere, midline shift, maximum DC diameter, time to decompression, anisocoria or bilaterally dilated pupils before DC, INR, aPTT, platelet count, whether the patients received anticoagulant, antiplatelet drugs, or both after CTS and before DC, and the occurrence of a new or progressive hemorrhagic lesion after DC. The predictor analysis used the data of 26 patients that had a reported mRS at 3–6 months. Since the predictor variables also exhibited some missing data (17 patients with complete data; 7.7% of all data was missing) we used multiple imputation to be able to use data from cases with incomplete reports (k = 30 imputations, R package “mice”)^19^. Due to the small sample size and many dichotomous predictors, we ran Bayesian logistic regression in the package: “brms”^20^. Twenty Markov Chain Monte Carlo samples with 10,000 iterations each were applied. A normal prior with mean 0 and standard deviation of 10 was used for all regression coefficients.

## Results

### Patient characteristics

**Figure 1:**
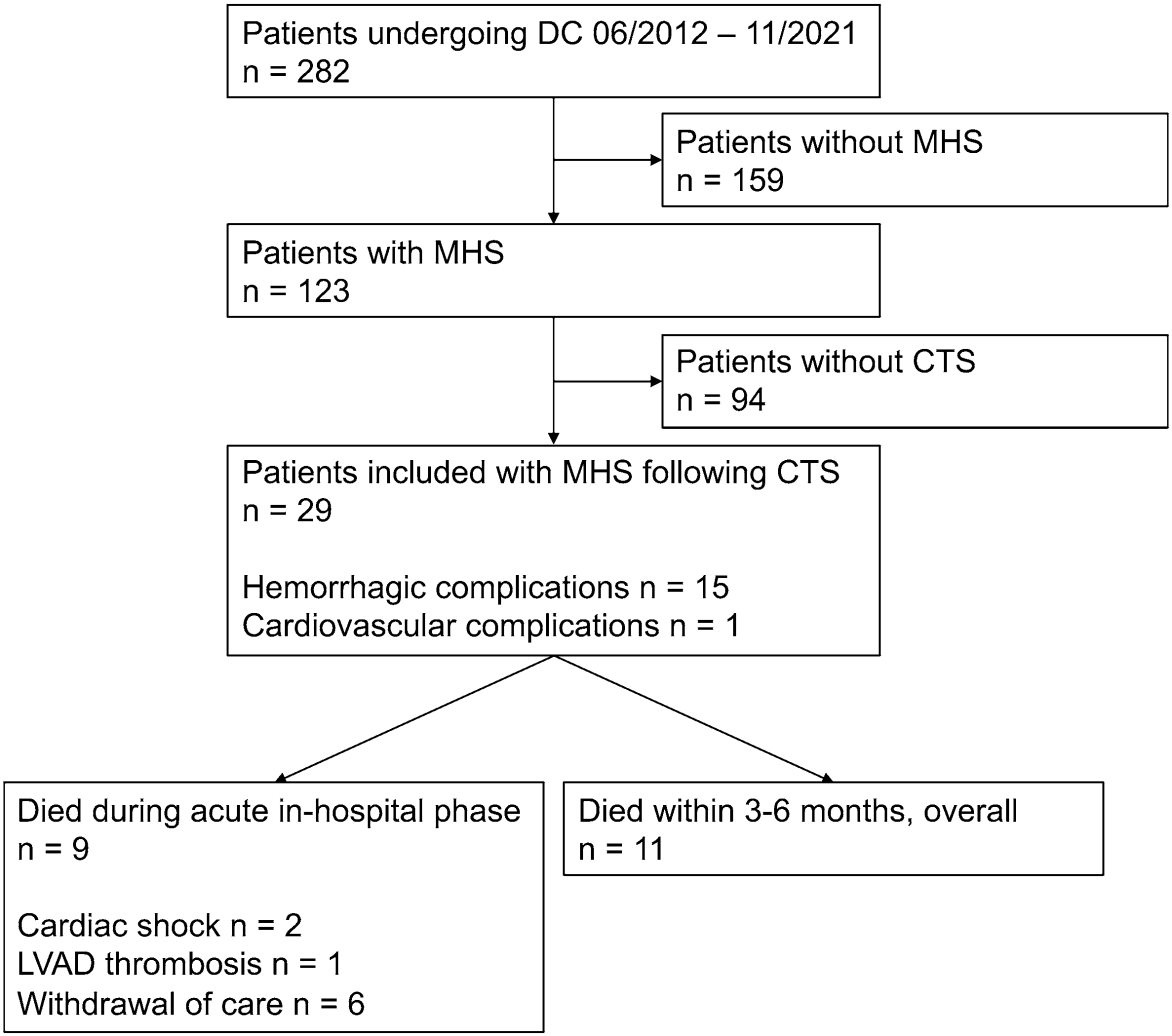
Flow diagram for included patients. *CTS* cardiothoracic surgery, *DC* decompressive hemicraniectomy, *LVAD* left ventricular assist device, *MHS* malignant hemispheric stroke, *n* number.

Overall, twenty-nine patients were identified (Fig. 1). Detailed information on patient characteristics and co-morbidities are shown in Tables 1 and 2. Most patients suffered type A or B acute aortic dissection (14/29, 48%) or underwent surgery for aortic or mitral valve repair or replacement (10/29, 34%). After CTS, 21/29 patients (72%) presented with inadequate recovery from anesthesia and 12/29 patients (41%) with hemiparesis or hemiplegia. Further, 2/29 (7%) had an additional generalized seizure and 12/29 (41%) a unilateral or bilateral mydriasis. The median time from diagnosis of MHS to DC was 2.6 (2.0–3.7) hours.

**Table 1:** Patient characteristics and outcome.

Patient no.	Sex, age	Cardiac pathology	MHS territory	Infarct vol., cm^3^, side	Infarct vol., corrected, cm^3^	Postop midline shift, mm	Time to DC, hours	DC diameter, mm	mRS 1 month	mRS 3–6 months
1	M, 64	TAAAD	ACA/MCA	730, R	587	1.2	2.4	181	5	6
2	F, 71	TAAAD	ACA/MCA	352, L	251	0.5	5.3	146	5	5
3	M, 62	TAAAD	MCA	392, R	336	8.8	11.4	167	4	4
4	F, 57	AVR	MCA/PCA	369, L	300	3.4	2.6	165	5	4
5	F, 61	CAB	MCA	198, R	176	3.5	2.9	158	5	5
6	M, 49	TAAAD	ACA/MCA	629, R	510	4.9	3.5	162	5	5
7	M, 59	LVAD implantation	ACA/MCA	478, L	381	10.2	2.2	156	6	6
8	M, 46	TAAAD	MCA	621, R	403	5.5	0.9	159	5	4
9	M, 66	PTCA	MCA	430, R	319	4.4	4.8	170	6	6
10	F, 33	TAAAD	MCA/PCA	347, R	304	0.6	1.3	166	5	4
11	M, 48	TAAAD	MCA	332, L	274	4.0	2.8	166	5	5
12	M, 63	MVR	MCA	350, R	289	2.8	3.0	167	5	n/a
13	F, 27	Aortic rupture	MCA	307, L	281	3.5	2.6	163	5	n/a
14	F, 62	TAAAD	MCA	496, L	380	2.4	3.1	173	5	4
15	M, 60	TAAAD	ACA/MCA	606, R	507	4.1	3.7	163	5	5
16	M, 51	MVR	MCA	483, R	387	4.3	1.5	164	6	6
17	M, 71	TAAAD	MCA	271, L	246	0.9	5.9	163	5	4
18	F, 64	TAAAD	ACA/MCA	464, L	356	8.6	2.0	157	6	6
19	M, 64	AVR	ACA/MCA	730, L	610	6.8	2.8	165	6	6
20	F, 61	MVR	MCA	243, L	212	3.2	1.6	157	5	3
21	M, 64	MV Repair	MCA	328, R	301	4.4	2.4	151	5	n/a
22	M, 50	TAAAD	MCA	442, R	359	5.6	2.2	157	6	6
23	F, 44	MVR	MCA	325, L	254	19.2	20.8	152	6	6
24	M, 40	AVR	MCA	454, L	343	0.5	2.3	156	6	6
25	M, 50	Ventricular Septal Rupture	ACA/MCA/PCA	n/a, R	n/a	n/a	3.9	162	5	6
26	F, 50	TBAAD	ACA/MCA/PCA	421, L	287	0	1.4	160	5	5
27	M, 61	AV repair	ACA/MCA	643, L	494	13.6	1.0	162	6	6
28	M, 68	AVR	MCA	243, L	209	0	16.0	153	5	5
29	M, 37	TAAAD	MCA	364, R	289	2.6	1.3	156	5	3
median (IQR)				407 (332–481)	312 (280–383)	4 (2–6)	2.6 (2.0–3.7)	162 (157–165)	5 (5–6)	5 (4–6)

*ACA* anterior cerebral artery, *ACVB* aortocoronary saphenous vein bypass, *AV* aortic valve, *AVR* aortic valve replacement, *CAB* coronary artery bypass, *DC* decompressive hemicraniectomy, *L* left, *LVAD* left ventricular assist device, *MCA* middle vertebral artery, *mRS* modified Rankin Scale, *MV* mitral valve, *MVR* mitral valve replacement, *n/a* not available, *PCA* posterior cerebral artery, *PTCA* percutaneous transluminal coronary angioplasty, *R* right, *TA(B)AAD* type A (B) acute aortic dissection, *vol.* *volume.*

**Table 2:** Preexisting co-morbidities.

	n = 29
Atrial fibrillation	8 (28%)
Arterial hypertension	9 (31%)
Diabetes mellitus type 2	5 (17%)
Pulmonary hypertension	3 (10%)
Renal failure	7 (24%)
Coronary heart disease	9 (31%)
Occlusive peripheral artery disease	2 (7%)
Heart failure	9 (31%)
Myocardial infarction	5 (17%)
Chronic endocarditis	2 (7%)
Coronary bypass surgery	3 (10%)
Valve surgery	2 (7%)
Coronary stent	3 (10%)
ICA- / aortic stent	2 (7%)
Ventricular assist device	2 (7%)
CRT-D	3 (10%)
Cerebral stroke	3 (10%)
Pulmonary embolism	4 (14%)
Hypothyreosis	4 (14%)
Rheumatic disease	4 (14%)
Marfan syndrome	2 (7%)
Cancer	4 (14%)
Other	7 (24%)

*CRT-D* Cardiac resynchronization therapy-defibrillator, *ICA* internal carotid artery, *IQR* interquartile range.

### Outcome

The in-hospital mortality rate was 31% and all 9/29 patients died within 4 (2–7) days after DC. Two patients died due to a cardiogenic shock with consecutive multiorgan dysfunction and 1 patient died due to a relapsing LVAD thrombosis. The remaining 6 patients died after withdrawal of care, following consultation with their family or next of kin and under consideration of the patient’s living will: In 1/6 patients, the decision to discontinue treatment was made after the clinical situation was further aggravated by septic shock. The other 5/6 patients were hemodynamically stable. One out of five had a patient decree regarding prolonged intubation/ventilation and refusing further treatment under consideration of the expected long-term dependency. In the remaining 4 patients, treatment was discontinued due to extensive postoperative hemorrhage, which further limited the already unfavorable prognosis.

Overall, 25/29 patients (86%) suffered from non-surgical and 7/29 patients (24%) from surgical complications after DC, which except for 3 patients that died from a cardiogenic shock or LVAD thrombosis likely had no effect on outcome (Table 3). All patients were transferred to a neurointensive care unit and back to a cardiac ICU or to neurorehabilitation after 26 (13–35) days. Among the 20 patients who survived the acute phase, tracheostomy was performed in 15/20 patients (75%) and 15/20 patients (75%) received cranioplasty after 98 (59–102) days. Three out of 20 patients (15%) required a ventriculoperitoneal shunt 121 (53–185) days after DC.

**Table 3:** Postoperative surgical and non-surgical complications.

	n = 29
Non-surgical complications	25 (86%)
Acute kidney failure	8 (28%)
Pneumonia, other infection	16 (55%)
Cardiogenic shock	4 (14%)
Septic shock	1 (3%)
Thrombosis or pulmonary embolism	10 (34%)
Progressive pleural effusion or hemothorax	8 (28%)
Meningitis or intracranial infection	6 (21%)
Other	7 (24%)
Surgical complications	7 (24%)
Surgical revision	
Wound healing complication	6 (21%)
Postoperative subgaleal hemorrhage	1 (3%)

**Figure 2:**
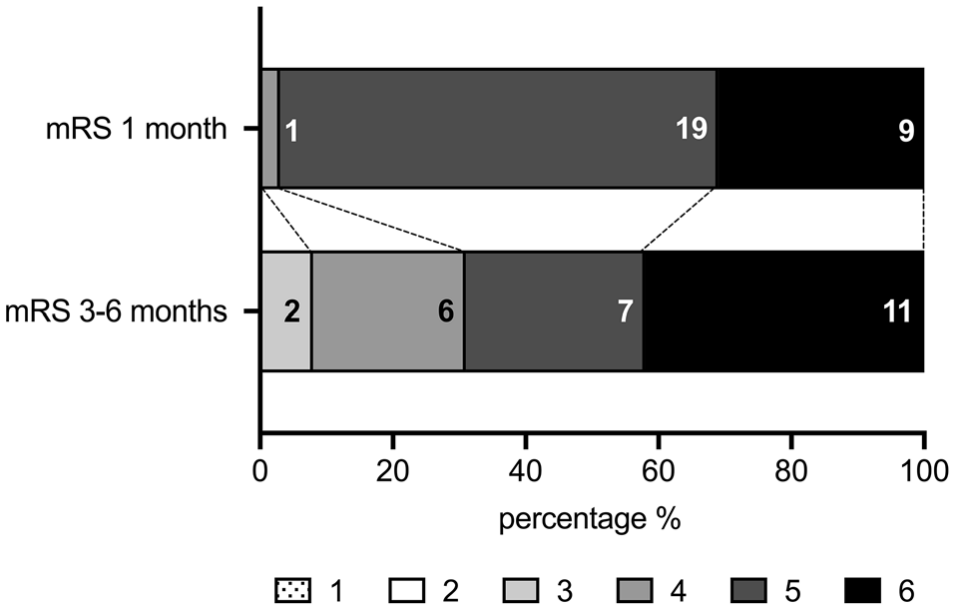
Development of neurological outcome. Distribution according to the modified Rankin Scale score at 1 months and at 3–6 months after decompressive surgery. The numbers inside the bar graphs represent the corresponding absolute number of events. *mRS* modified Rankin Scale.

At 1-month, the median mRS of 29 patients was 5 (5–6), remained stable at 3–6 months with an mRS of 5 (4–6). At this time point, the overall mortality rate was 42% (11/26 patients). Three out of 29 patients (10%) were lost to follow-up after 3–6 months (Fig. 2).

### Anticoagulation management

**Figure 3:**
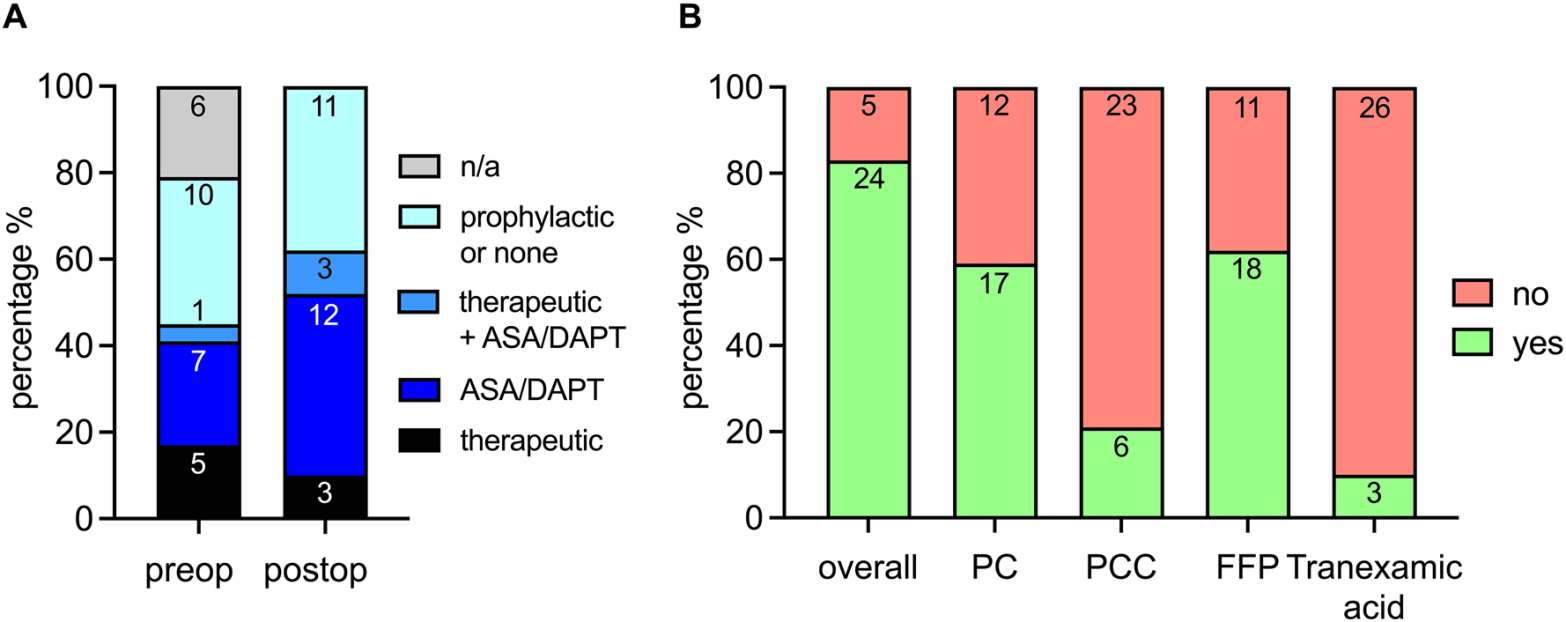
Perioperative anticoagulation/platelet inhibition and substitution. (**A**) Pre- and postoperative anticoagulation and platelet inhibition before and after decompressive hemicraniectomy. (**B**) Perioperative substitution of coagulations factors and platelets during decompressive hemicraniectomy. The numbers inside the bar graphs represent the corresponding absolute number of events. *ASA* acetylsalicylic acid, *DAPT* dual antiplatelet therapy, *FFP* fresh frozen plasma, *PC* platelet concentrate, *PCC* prothrombin complex concentrate.

Before DC, 23/29 patients (79%) had received anticoagulation and/or antiplatelet therapy (Fig. 3A): 12/29 patients (41%) received heparin and 2/29 (7%) argatroban only, 1/29 patients (3%) received phenprocoumon, 4/29 patients (14%) received heparin and acetylsalicylic acid (ASA), 1/29 patients (3%) received heparin and clopidogrel, 1/29 patients (3%) received ASA and ticagrelor and 2/29 patients (7%) received ASA alone. For the other 6/29 patients (21%), documentation on preoperative anticoagulation and antiplatelet therapy was not available. Overall, only 6/29 patients (21%) reached therapeutic anticoagulation within an aPTT of 50–70 s or INR > 2 before DC and uncorrected coagulation parameters after CTS and before DC were within normal ranges [Quick (prothrombin time) 69 (56–76) %; INR 1.3 (1.2–1.5); aPTT 39 (37–47) seconds]. The median platelet count was 135 (106–181)/nl.

Pre- or intraoperatively, 24/29 patients (83%) received substitution with platelets, fresh frozen plasma, tranexamic acid, fibrinogen or prothrombin complex (Fig. 3B). Twenty out of 29 patients (69%) received red blood cell concentrates.

Postoperatively, 11/29 patients (38%) received no or only prophylactic anticoagulation with heparin or argatroban, 3/29 patients (10%) received therapeutic anticoagulation, 12/29 patients (42%) received antiplatelet therapy with ASA and/or Clopidogrel, and 3/29 patients (10%) received both therapeutic anticoagulation and antiplatelet therapy (Fig. 3A). In 18/29 patients (62%), therapeutic anticoagulation and/or antiplatelet therapy was re-initiated 6 (4–11) days after DC.

### Postoperative thrombotic cardiovascular and hemorrhagic intracranial complications

Fifteen out of 29 patients (52%) presented a postoperative intracranial hemorrhage on postoperative day 1 (1–5). Four of these 15 patients (27%) suffered a massive ICH on postoperative day 1 (1–2) despite perioperative substitution. Of note, all but one new hemorrhagic lesion occurred before the discontinued anticoagulation and/or platelet inhibition were re-initiated. Bayesian logistic regression yielded a longer preoperative aPPT and new or progressive postoperative hemorrhagic lesions on CT imaging as statistically significant predictors of mortality (p < 0.05; Table 4). In complete data sets, both variables displayed strongly increased risks when analyzed bi-variately: If aPPT was prolonged by ten seconds, the risk of mortality increased 14-fold (OR = 13.94, p = 0.038). If a new or progressive lesion after DC was noted, the risk of mortality was tripled (OR = 3.03, p = 0.19).

**Table 4:** Predictors of mortality (mRS = 6) in Bayesian logistic regression after multiple imputation (n = 26).

	*b*	Lower 95%-CI	Upper 95%-CI
Age	0.02	− 2.06	2.64
Sex (male)	− 0.21	− 18.24	17.81
Emergency CTS (yes)	− 9.49	− 27.49	8.34
TA(B)AAD surgery (yes)	− 13.12	− 30.57	4.26
Valve Surgery (yes)	9.29	− 8.42	27.35
Hours to DC	− 0.17	− 0.97	0.66
Size of DC	2.23	− 0.93	6.05
Infarct volume	0.04	− 0.12	0.20
Midline shift	0.54	− 0.04	1.24
aPTT	**3.72**	**0.14**	**9.89**
INR	− 3.92	− 23.54	15.72
Dilated Pupil (yes)	8.54	− 8.68	25.96
Hemisphere (left)	10.72	− 6.58	28.58
New lesion (yes)	**17.10**	**2.06**	**32.97**
Platelet count	− 0.18	− 0.58	0.08
Anticoagulation (yes)	1.53	− 17.11	20.28
Antiplatelets (yes)	− 1.06	− 19.28	17.29
Anticoagulation AND antiplatelets (yes)	0.81	− 18.47	19.96

*b* Bayesian regression coefficient, *CI* confidence interval, *CTS* cardiothoracic surgery, *DC* decompressive hemicraniectomy, *aPTT* activated partial thromboplasty time, *INR* International Normalized Ratio, *mRS* modified Rankin Scale, *TA(B)AAD* type A (B) acute aortic dissection

Bold font indicates statistical significance at *p* < 0.05.

Despite perioperative discontinuation of anticoagulation and/or platelet inhibition, no coagulation-associated cardiovascular complications were noted, except for a recurrent LVAD thrombosis in a patient who already had suffered an initial pump thrombosis immediately after LVAD implantation and underwent an endovascular thrombectomy.

### Missing patient data

Documentation of the preoperative anticoagulation and/or antiplatelet therapy before DC was missing in 6/29 patients (21%). Preoperative coagulation parameters before DC were not available in 1/29 patients (3%). Documentation of perioperative coagulation factor and platelet substitution was missing in 1/29 patients (3%). Three out of 29 patients (10%) were lost for follow-up data at 3–6 months. In 1/29 patients (3%) no postoperative CT within 24 h was available for infarct volumetry.

## Discussion

In this study, we found that DC in patients with MHS following CTS may help reduce mortality compared to the natural course of MHS. Specifically, a prolonged aPTT and new or progressive postoperative hemorrhagic lesions were found to be associated with a significantly greater risk for mortality at 3–6 months. This highlights the need to optimize preoperative coagulation, because even after discontinuation and correction of anticoagulation and/or platelet inhibition, the risk for cardiovascular complications remained low. Nevertheless, the benefit of reducing mortality through decompressive surgery needs to be carefully balanced against a high likelihood of unfavorable neurological outcome.

Malignant hemispheric stroke is a life-threatening condition with a mortality of up to 80% in intensive care-based series^21^. Randomized controlled trials (RCTs) performed in MHS patients without significant comorbidities have evidenced that early DC undertaken within 48 h of stroke onset significantly reduces mortality^10,12–14^. Unfortunately, early identification of patients with neurological deterioration after CTS remains challenging due to the prolonged recovery phase from anesthesia. Although extended neuromonitoring might allow early identification of patients with space-occupying perioperative stroke following CTS^22,23^, in clinical practice, monitoring is often limited to neurological examination and affected patients are mainly identified by prolonged impaired consciousness, hemiplegia, seizures or dilated pupils due to an already manifest and life-threatening space-occupying brain edema. Apart from this delayed detection, another problem regarding decision-making and patient counselling is that CTS patients suffer serious cardiovascular comorbidities. Therefore, the known randomized controlled trials on MHS cannot be generally translated to this patient population^14^.

A natural conflict exists between coagulation requirements following CTS on one side, and after DC for MHS on the other, where the main goal is to limit the risk of postoperative hemorrhage and secondary lesion progression by strict anticoagulation control and hemodynamic management^22^. To the best of our knowledge, only few case reports exist regarding the feasibility but not functional outcome of performing DC in the context of acute aortic repair^24–27^ or LVAD thrombosis^28^. Against this background, the present study represents the first and largest series to date and aims to provide comprehensive information regarding outcome and coagulation management in patients undergoing DC after CTS.

### Mortality and functional outcome

In the present study, the overall mortality rate of 42% after 3–6 months appears to be lower than the mortality rate of up to 80% that has been reported for the natural course of MHS in patients without severe cardiothoracic comorbidity^21^. On the other hand, mortality was also higher than the 29–33% described in the known RCTs on MHS (DESTINY, DECIMAL and HAMLET)^14^ but these trials had excluded patients with a pre-morbid mRS > 1, pre-morbid Barthel-Index < 95, and with other concomitant severe diseases that would likely have affected treatment or reduced life expectancy below 3 years. In contrast, patients in our study population suffered severe pre-existing cardiovascular co-morbidities and underwent a major CTS procedure, which may likely affect morbidity and mortality. This is mirrored by the fact that in our cohort only 7% reached an mRS score of 3 and none had an mRS ≤ 2 after 3–6 months, compared to previously published RCTs where favorable outcome (mRS 0–3) was reached in 25–47% after 6 months^10–14^. Therefore, our present results rather compare to MHS patients with a median age of 70, where DC may still reduce mortality (33% vs. 70% without surgery), but with a much lower likelihood of merely 6% that experience favorable outcome (mRS 0–3) at 6 months^11^.

Another prognostic factor is the time between stroke onset and DC. Latest studies suggest that DC performed after 48 h after stroke onset might still have a beneficial effect on mortality and outcome and that herniation could be the more decisive factor, rather than the mere temporal aspect^29^. Due to the delayed recovery from anesthesia, however, the stroke onset in patients after CTS is difficult to determine. In fact, in 41% of our patients the decision to perform CT imaging was triggered by uni- or bilaterally dilated pupils due to an already existing, space-occupying brain edema, which highlights that patients in our cohort were more severely affected than patients included in previous RCTs and this could at least partially explain why the benefit of survival that we observed was associated with a strikingly high proportion of patients that experienced severe neurological disability. Next to other factors^22,30,31^, this risk of severe disability must be taken into account when counselling patients and caregivers on what to expect from decompressive surgery after CTS The delayed identification of patients with postoperative stroke following CTS further emphasizes the need for continuously improving interdisciplinary standard operating procedures for stroke detection, for example by implementing routine CT perfusion imaging after certain high-risk CTS procedures as a tool for earlier stroke diagnosis.

### Postoperative anticoagulation management and complications

Approximately 79% of the patients in our study had received anticoagulant and/or antiplatelet therapy before DC but only 21% had therapeutic aPTT levels. This discrepancy is partially explained by the fact that the effect of antiplatelet medication is not reflected by anticoagulation parameters. Further, the short interval between CTS and DC may have additionally hampered an establishment of stable heparin levels^32^. Nevertheless, we observed a significant association between a prolonged aPTT and the development of new or progressive hemorrhagic lesions and the overall mortality after DC, which suggests an increased risk related to anticoagulation after CTS and highlights the importance of optimizing coagulation parameters before DC. Under perioperative substitution, the risk of severe space-occupying ICH was still 14% but this appears to be in line with the approximately 10% rate of new ipsilateral hemorrhagic complications reported in the literature across all indications for DC^33,34^. Thus, the risk for severe postoperative hemorrhagic complications after DC may be considered to be acceptable, given the severity of the disease and complex anticoagulation management requirements in CTS patients. This is further supported by the fact that the vast majority of postoperative hemorrhagic lesions were noted *before* a re-initiation of anticoagulation and/or platelet inhibition was begun. Most importantly, despite perioperative substitution and discontinuation of anticoagulation and/or platelet inhibition, no thromboembolic cardiovascular complications occurred, except for one recurrent pump thrombosis in a patient who had already suffered an initial pump thrombosis immediately after LVAD implantation. The high rate of cranioplasty in our study despite the high proportion of patients with unfavorable outcome remains debatable and the management of anticoagulant/antiplatelet therapy adds another layer of complexity to the cranioplasty decision-making process. However, several reports have shown clinical improvement after cranioplasty, suggesting that the craniectomy defect itself may prolong recovery, especially in patients with a sunken skin flap and syndrome of the trephined^35,36^. Thus, cranioplasty might facilitate neurological rehabilitation regardless of the degree of disability, next to providing protection of the underlying brain during mobilization and nursing activities^37^. Together with the low rate of thromboembolic complications that we experienced, we believe that the consideration of performing cranioplasty is also justified in a high-risk CTS population.

### Limitations

This study bears well known limitations due to its retrospective design and limited patient number and our database only provided data on functional outcome until 3–6 months after the event. On the other hand, DC in CTS remains rare the short follow-up period appears reasonable, considering the severe co-morbidities within our cohort. The major limitation of our study is the missing control group of CTS patients with MHS that were managed without DC. This lacking control most likely mirrors our own selection bias following the ethical consideration that in situations of MHS after CTS where survival is already judged to be highly unlikely at the time-point of diagnosis, the reasonable interdisciplinary decision was made to rather offer supportive care than surgical decompression.

## Conclusion

Cardiothoracic surgery patients suffering MHS will likely suffer severe neurological disability after DC, since the majority of patients in our study experienced either mortality or significant long-term disability. This should remain a central aspect during counselling and decision-making and given the poor prognosis, surgeons need to carefully consider whether DC should be performed at all. Nevertheless, the fact that we observed no thromboembolic complication due to discontinuation of anticoagulation and/or antiplatelet therapy argues that the complex coagulation situation after CTS should not per se rule out the option of performing life-saving surgical decompression.

## Data Availability

The datasets used and analyzed during the current study are available from the corresponding author on reasonable request.
